# Epidemic History of Hepatitis C Virus among Patients with Inherited Bleeding Disorders in Iran

**DOI:** 10.1371/journal.pone.0162492

**Published:** 2016-09-09

**Authors:** Katayoun Samimi-Rad, Ramin Rahimnia, Mahdi Sadeghi, Seyed Amir Malekpour, Mona Marzban, Maryam Keshvari, Seyed Jalal Kiani, Seyed-Moayed Alavian

**Affiliations:** 1 Department of Virology, School of Public Health, Tehran University of Medical Sciences (TUMS), Tehran, Iran; 2 Department of Nano medicine, School of Advanced Technologies in Medicine, TUMS, Tehran, Iran; 3 National Institute of Genetic Engineering and Biotechnology, Tehran, Iran; 4 School of Mathematics, Statistics and Computer Science, College of Science, University of Tehran, Tehran, Iran; 5 Blood Transfusion Research Center, High Institute for Research and Education in Transfusion Medicine, Tehran, Iran; 6 Research Center for Gastroenterology and Liver Disease, Baqiatallah University of Medical Sciences, Tehran, Iran; National and Kapodistrian University of Athens, GREECE

## Abstract

The high rate of hepatitis C virus (HCV) infection among transfusion related risk groups such as patients with inherited bleeding disorders highlighting the investigation on prevalent subtypes and their epidemic history among this group. In this study, 166 new HCV NS5B sequences isolated from patients with inherited bleeding disorders together with 29 sequences related to hemophiliacs obtained from a previous study on diversity of HCV in Iran were analyzed. The most prevalent subtype was 1a (65%), followed by 3a (18.7%),1b (14.5%),4(1.2%) and 2k (0.6%). Subtypes 1a and 3a showed exponential expansion during the 20^th^ century. Whereas expansion of 3a started around 20 years earlier than 1a among the study patients, the epidemic growth of 1a revealed a delay of about 10 years compared with that found for this subtype in developed countries. Our results supported the view that the spread of 3a reached the plateau 10 years prior to the screening of blood donors for HCV. Rather, 1a reached the plateau when screening program was implemented. The differences observed in the epidemic behavior of HCV-1a and 3a may be associated with different transmission routes of two subtypes. Indeed, expansion of 1a was more commonly linked to blood transfusion, while 3a was more strongly associated to drug use and specially IDU after 1960. Our findings also showed HCV transmission through blood products has effectively been controlled from late 1990s. In conclusion, the implementation of strategies such as standard surveillance programs and subsiding antiviral treatments seems to be essential to both prevent new HCV infections and to decline the current and future HCV disease among Iranian patients with inherited bleeding disorders.

## Introduction

The history of blood transfusion in Iran dates back to the 1940s. However, in 1952, a blood transfusion center was founded by the former “Red Lion and Sun Society" (later named the Red Crescent Society) [1]. In 1961, this Society started providing locally single blood donor products. At the same time, a military blood bank was also established[2]. Up to the late 1960s, all of these centers relied exclusively upon paid donors, most of them were drug-addicted. The Iranian Blood Transfusion Organization (IBTO) was formally established in 1974, through which all blood transfusion activities were centralized including donor recruitment, production of blood components, and delivery of blood and blood products[3]. Although blood units in Iran were screened for common infectious agents, HCV screening started from 1996. Over the period of 1970 to 1986, the majority of patients with hemophilia were treated with locally provided fresh frozen plasma and cryoprecipitate. However, a small proportion of these patients were sometimes receiving untreated factor concentrates. Non-virally inactivated factor concentrates were only produced between 1994 and 1997 in Iran[4].

Several reports on Iranian patients with inherited bleeding disorders have used Inno Lipa assay or genotype specific primers to determine HCV genotypes and subtypes [5, 6]. These methods do not often provide accurate information at the subtype level. However, studies using direct DNA sequencing and phylogenetic analysis based on HCV genomic regions with suitable variability such as NS5B and core further provided a definitive genetic classification[7].

In recent years, molecular epidemiological methods are used to estimate the epidemic history of HCV in some countries [8–10]. These methods were mostly based on coalescent theory and Bayesian Skyline Plot (BSP) can reconstruct the past transmission history and population dynamics of HCV subtypes from viral gene sequences. Analysis of these data can indicate which subtypes are growing in an exponential or constant rate and may shed light on their future incidence in the study populations. Furthermore, these analyses may also have important clinical implications particularly in HCV treatment and effective strategies to prevent the spread of HCV infection.

The present study aims to both determine the HCV genotype distribution among Iranian patients with inherited bleeding disorders based on sequencing of NS5B region followed by phylogenetic analysis and to conduct a molecular study on these NS5B sequence data. Our results revealed a history of epidemic spread of these isolates within this high risk group. They also supported an evidence for the plausible role of new transmission networks in exponential growth of subtypes1a and 3a during the 20^th^ century in these patients.

## Material and Methods

### Study Patients

A total of 229 anti-HCV positive serum samples were collected from patients with inherited bleeding disorders at a major referral clinic in Tehran. For all subjects, demographic, laboratory and clinical features for HCV infection were obtained via questionnaire and patient profile. All patients or their parents (in the case of pediatric subjects) signed an informed consent before sampling. The study protocol was approved by ethical committee in Tehran University of Medical Sciences in accordance with the Helsinki Declaration. These subjects included 215 males and 14 females with the mean age 30.6 ± 10.59 years (median 28; range 13–70). The highest rate of these patients was observed in the 21-to 30-year age group. Among the participants, 69% had hemophilia A, 12.6% hemophilia B, and 18.4% other inherited bleeding disorders. While, one hundred and sixty (59.4%) of these subjects had severe hemophilia disease, 66 (28.8%) had non-severe disease. For 27(11.8%) of these patients severity of disease was unknown. The majority (n = 225, 98.3%) of the study patients were negative for HBsAg. The mean duration of treatment (the time of first transfusion with blood products up to the time of sampling) was 300.23 ± 101.95 months. Fifty one (23.7%) males and one (7.1%) female had normal SGPT levels (≤30 U/L and ≤20 U/L for males and females, respectively). Among the participants, 221 (96.5%) started receiving blood or blood products before 1996 (the year of implementation of blood donor screening for HCV in Iran) and the remaining 8 (3.5%) after 1996. These patients were mainly treated with clotting factor concentrates (n = 202, 88%), cryoprecipitate (n = 187, 81.7%), fresh frozen plasma (n = 154, 67.2%) and packed cell (n = 101, 44.1%). All patients were negative for HIV.

### RNA Extraction and PCR

HCV RNA was extracted from 140μl of each serum sample using the QIAmp viral RNA extraction Kit (QIAGEN, Valencia, CA) according to the manufacturer's protocol. HCV RNA sequences were amplified within the NS5B region forming a 377 bp product using the QIAGEN One Step RT-PCR Kit. The NS5B amplicons were purified from the PCR reaction by a PCR Purification Kit (QIAGEN) and then sequenced directly (Eurofins MWG Operon, Germany).

### Sequence Data

We included 29 partial NS5B HCV sequences from hemophilia patients, which were obtained from a previous report of HCV diversity in Iran[11]. These sequences were aligned with the newly 166 generated sequences from patients with inherited bleeding disorders. In total, the resulting NS5B alignment contained 195 sequences.

### Nucleotide sequence accession numbers

The Gene Bank accession numbers of the 166 new HCV sequences from patients with inherited bleeding disorders are KC351575 to KC351742.

### Phylogenetic analysis

167 reference sequences of NS5B gene related to the three major HCV subtypes including 1a,1b and 3a, were downloaded from GenBank and aligned using ClustalX 2.1 along with our sequences obtained in this study.

For each subtype sequences, the most appropriate nucleotide substitution model was selected using JModelTest -2.1.4, assessed by the Akaike Information Criterion[12, 13]. While HKY+Г+I was the best-fitting model for both subtypes 1a and 1b, TrN93+Г+I was found to be the best model for subtype 3a. The phylogenetic tree of each subtype sequences was constructed by the maximum likelihood methodology with PhyML 3.0[14, 15]. To assess the reliability of the phylogenetic trees, boot strap resampling tests were carried out in 1000 replications. The trees were prepared for publication using Fig Tree v 1.4.0 0 (http://tree.bio.ed.ac.uk/software/figtree).

### Evolutionary analysis of NS5B data set in Iranian patients with inherited bleeding disorders

Three phylogenetic trees containing clusters with high bootstrap values were construced. In addition, five clusters were analysed, including cluster A and B which contain 90 and 5 subtype 1a sequences, respectively, cluster C which contains 14 subtype 1b sequences, and cluster D and E which contain 11 and 8 subtype 3a sequences, respectively. For these clusters, evolutionary parameters, time to Most Recent Common Ancestor (tMRCA) and population dynamics of infections were estimated using Bayesian Monte Carlo Markov Chain Method (BMCMC) as implemented in the BEAST software, version 1.8.0, (http://evolve.zoo.ox.ac.uk/beast/).

To analyze each cluster, the most appropriate nucleotide substitution model was used. This model was selected using jModel Test-2.1.4.

Moreover, different rate heterogeneity parameters were given to each codon position. There was insufficient temporal structure in the study sequences for the direct estimation of the nucleotide substutution rate. Thus, we used previously estimated substitution rates of NS5B [6, 16, 17]for all subtypes to generate results on the time scale of years. For subtypes 1a and 3a, the substitutions rate per site per year was 3.68*10^−4^ while this rate for subtype 1b was 5*10^−4^. These rates were used to define normally prior distribution when we were carrying out the analyses for each cluster.

The three clock models that use the Bayesian MCMC approach implemented in the program BEAST were applied to estimate posterior distributions of these rates. These models include 1- strict clock 2- relaxed clock with an uncorrelated lognormal distribution, and 3- relaxed clock with an uncorrelated exponential distribution[8, 9]. The strict and relaxed clock analyses were performed for each cluster and the best fitting model based on marginal likelihood was chosen.

The point estimations (median) and confidence intervals for evolutionary parameters such as rate heterogeneity parameter, transition/transversion rate ratio and nucleotide substitution rates were obtained by the BMCMC for runs of 15 000 000 states, sampled every 3000 states. The Effective Sample Size (ESS) and convergence of the sampling was then monitored using Tracer software version 1.6 (http://tree.bio.ed.ac.uk). For this long MCMC sampling, ESSs usually were higher than 300 for all parameters. In the case that ESSs were not large enough, we applied a longer MCMC sampling. Samples were also monitored visually to check for the stationarity in distribution.

Moreover, the demographic history and population dynamic of infections in the Iraninan HCV clusters were estimated using Bayesian Skyline Plot (BSP) as implemented in the program BEAST[10, 18]. The BSP provided estimations about the effective number of infections at every point in time back to MRCA, for clusters A, B, C, D, and E. Based on BSP results, the time intervals that effective number of infections had increased exponetially were identified. Then linear regression was used to estimate the viral exponetial growth rate from the BSP results. The coefficient of time in this linear regression was considered as the exponetial growth rate.

## Results

### Detection of HCV RNA and genotyping

Out of 229 positive samples for HCV antibody, 175(76.5%) had detectable HCV RNA. Among 175 positive samples for NS5B region, 166 were genotyped by sequencing. Subtype1a, 3a, and1b were predominant accounting for 65% (n = 108), 18.7% (n = 31), and 14.5% (n = 24), respectively. However, type 4 and subtype 2k were the least frequent accounting for 1.2% (n = 2) and 0.6% (n = 1), respectively.

### Phylogenetic analysis

**Fig 1:**
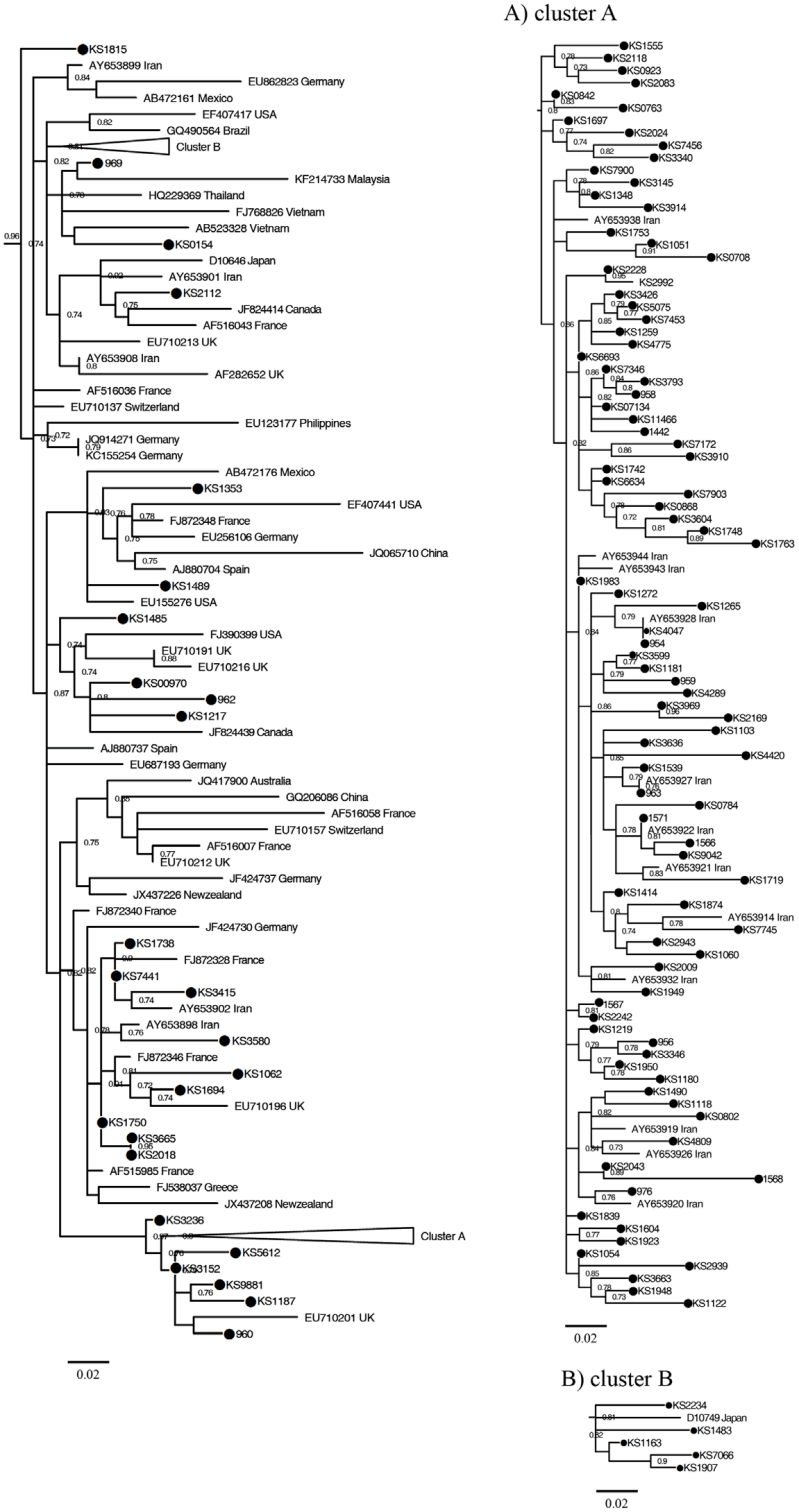
Phylogeny of subtype 1a strains and reference strains estimated from NS5B sequences. It obtained by maximum likelihood method using HKY+*Г*+I as nucleotides' substitution model. Trianglesshow the position of clusters A and B which are detailed in (A) and (B), respectively. The scale bars are in units of nucleotide substitutions per site. The number above/below the branches represents the bootstrap values. Accession number and origin of the reference strains are given at the nodes.

**Fig 2:**
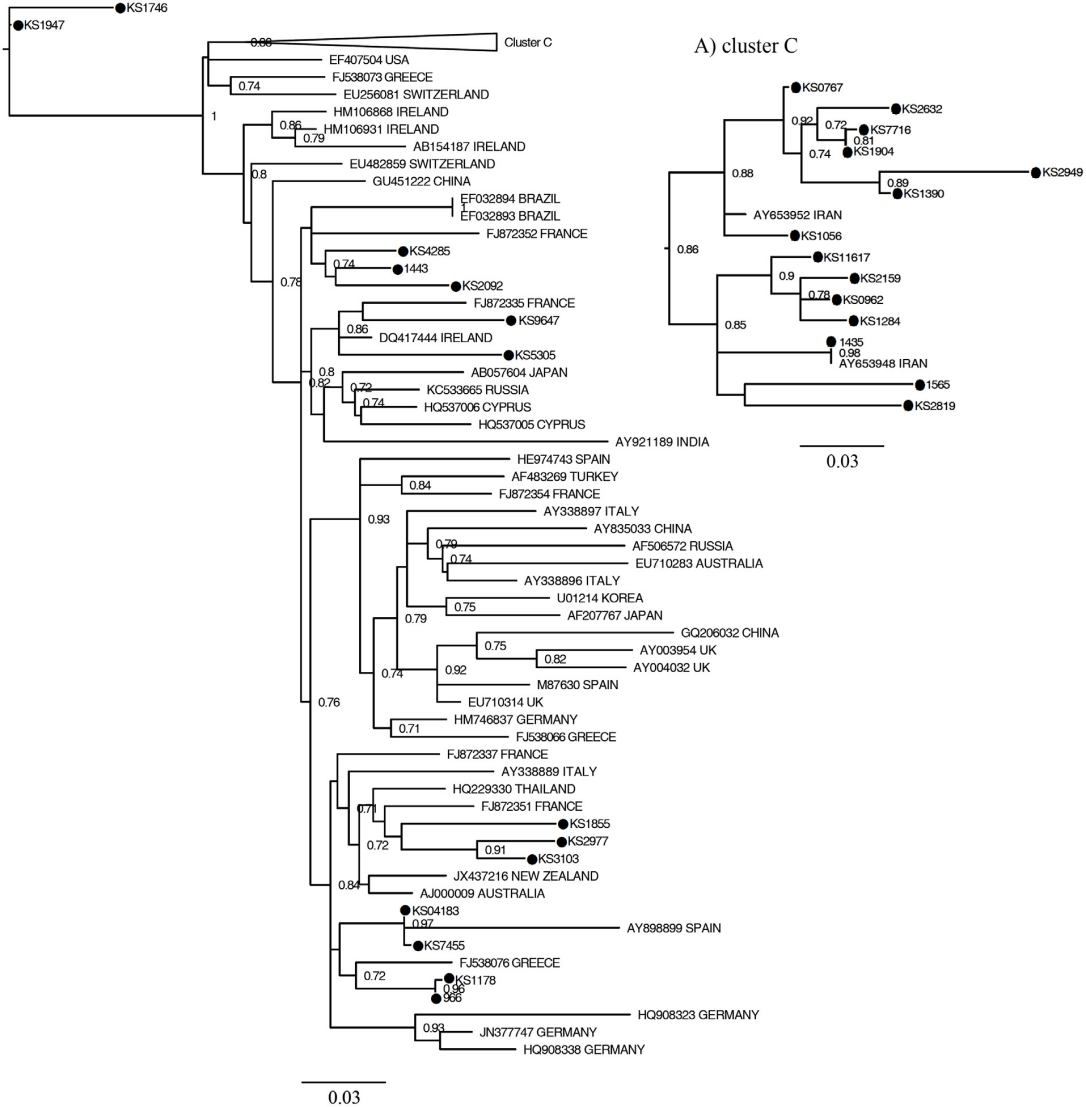
Phylogeny of subtype 1b strains and reference strains estimated from NS5B sequences. It obtained by maximum likelihood method using HKY+*Г*+I as nucleotides' substitution model. Triangleshows the position of cluster C which is detailed in (A). See Fig 1 legend for more details.

**Fig 3:**
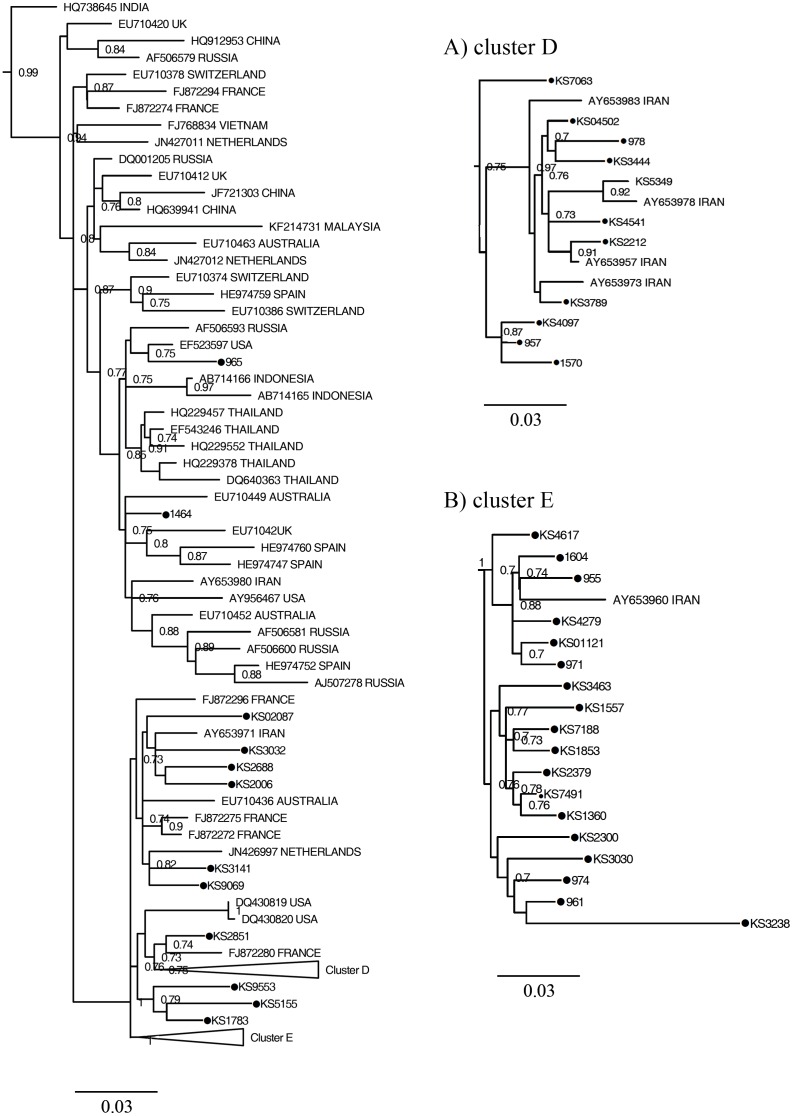
Phylogeny of subtype 3a strains and reference strains estimated from NS5B sequences. It obtained by maximum likelihood method using TrN93+*Г*+I as nucleotides' substitution model. Triangles show the position of cluster D and E which are detailed in (A) and (B), respectively. See Fig 1 legend for more details.

Figs 1, 2 and 3 show the maximum likelihood phylogenies estimated from the NS5B gene sequences including 166 sequences isolated from patients with inherited bleeding disorders in this study, 29 sequences obtained from Iranian hemophiliacs from a previous report and 167 reference sequences.

Out of 120 subtype 1a sequences, the 90(75%) strains formed cluster A where non-Iranian reference strains were not observed in this cluster (Fig 1A). Twenty five (20.8%) of the remaining subtype 1a strains were older than those of cluster A. The lower prevalence of the older strains compared to cluster A strains suggests that the proportion of older sequences has not grown through time and most of HCV-1a strains obtained from the study patients appear to introduce to Iran in a specific period of time in the past.

Only five out of 25 old strains formed the cluster B (Fig 1B) and the rest 20(16.6%) distributed among the reference sequences from other countries. Although this rate (16.6%) is much lower than that of the subtype 1b and 3a sequences, it suggests multiple sporadic introductions of subtype 1a strains into our study patients from other countries. The bootstrap values for cluster A and B were 80% and 82%, respectively.

The phylogenetic analysis of subtype 1b is shown in Fig 2. As indicated, 14 out of the 28 subtype 1b sequences formed cluster C together with Iranian reference strains and did not include reference sequence from any other country (Fig 2A). This cluster was branched from one of the oldest nodes in the phylogenetic tree. The remaining 14 subtype 1b strains dispersed among the reference sequences from other countries and have introduced to Iran more recently, suggesting that these sequences may have various origins compared to those of cluster C.

Forty one strains of patients with inherited bleeding disorders belonged to subtype 3a and within this subtype two clusters could be identified (clusters D and E) as shown in Fig 3. Cluster D was formed by 11 strains from patients with inherited bleeding disorders and 4 strains from Iranian reference sequences (Fig 3A). Eighteen other strains formed cluster E together with one isolate (Ay653960TD) from our Iranian reference sequences (Fig 3B). These two clusters were constructed with bootstrap values of 75% for cluster D and 100% for E. The remaining subtype 3a sequences were randomly intermixed with other reference 3a sequences. Most of these references were from other countries. Phylogenetic tree of subtype 3a indicates that strains of cluster E are older than cluster D.

### Evolutionary analysis of NS5B data set in Iranian patients with inherited bleeding disorders

The estimates of substitution rate parameters and population dynamics in four clusters were performed in BEAST under three molecular clock models [10, 19]. For each model, both BSPs with 5 and 10 steps were used.

These models were applied as (A) a strict clock with BSP of 5 steps, (B) a strict clock with BSP of 10 steps, (C) a log normal relaxed clock with BSP of 5 steps, (D) a log normal relaxed clock with BSP of 10 steps, (E) a gamma relaxed clock with BSP of 5 steps, and (F) a gamma relaxed clock with BSP of 10 steps.

**Fig 4:**
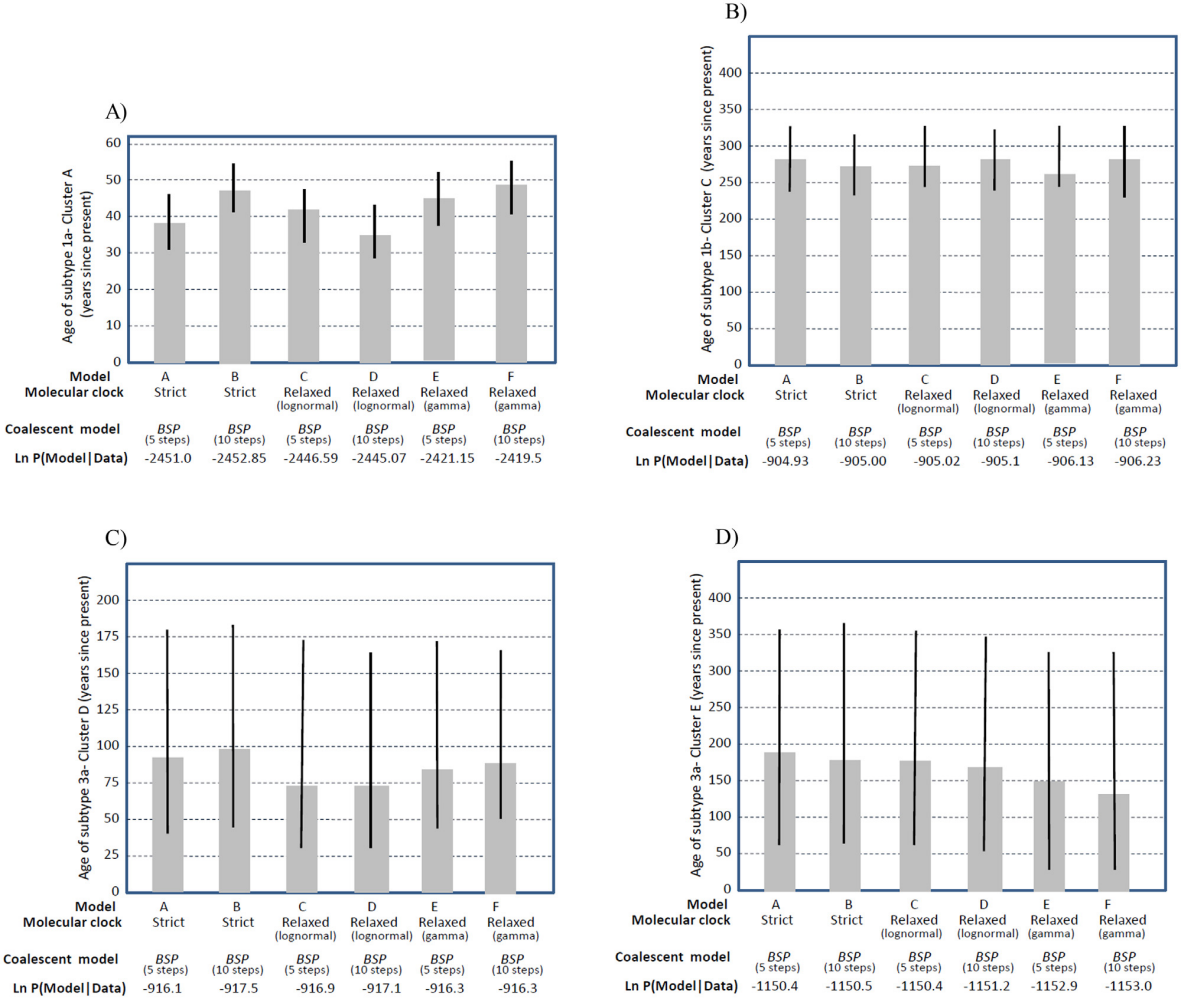
Estimated dates of HCV subtypes origin, as obtained under model combinations A to F. (A) Subtype 1a, cluster A. (B) subtype 1b, cluster B. (C) subtype 3a, cluster D. (D) subtype 3a, cluster E (see text for further details). The bars show the 95% credible intervals of each estimate.

The estimated marginal likelihood of each model is shown in Fig 4. For clusters C, D, and E, the model A had higher marginal likelihood than the other models, although the difference found, was not significant (log 10 bayes factor, <0.5) (Fig 4B, 4C and 4D). For this clock model, the BSP with 5 steps favored over the BSP with 10 steps. The six models in Fig 4b also showed the median estimates for the age of HCV subtype 1b which was dated to ~263 to 283 years ago. The models in Fig 4C and 4D gave median estimates for the two clusters of subtype 3a which were dated to ~ 73 to 98 and ~138 to 188 years ago, respectively.

For cluster A, the model F had significantly greater marginal likelihood than the remaining models (log 10 Bayes factors of > 3.5) (Fig 4A). However, there was no significant difference in marginal likelihood between model E and F. For clock model F, the BSP with 10 steps was favored over the BSP with 5 steps. Thus, models F and A were found to be the most appropriate models for evolutionary analysis of NS5B data set in Iranian patients with inherited bleeding disorders. The age of subtype 1a was dated to ~34 to 49 years ago based on the median estimates as indicated by models A to F (Fig 4A). The 95% credible intervals for these estimates in cluster A was small ranging from 29 years to nearly 56 years ago and in cluster C was larger ranging from 233 years to 333 years ago. These values for subtype 3a in cluster E were ranging from 33 years to nearly 363 years ago which was larger than that of cluster D with a range from 31 years to 185 years ago. Our estimates of the age of isolates in four clusters showed that subtype 3a strains in cluster E were the oldest and 1a strains in cluster A were the youngest HCV isolates among Iranian patients with inherited bleeding disorders, suggesting the greater diversity of isolates in cluster E compared with the strains of other clusters, particularly cluster A.

**Fig 5:**
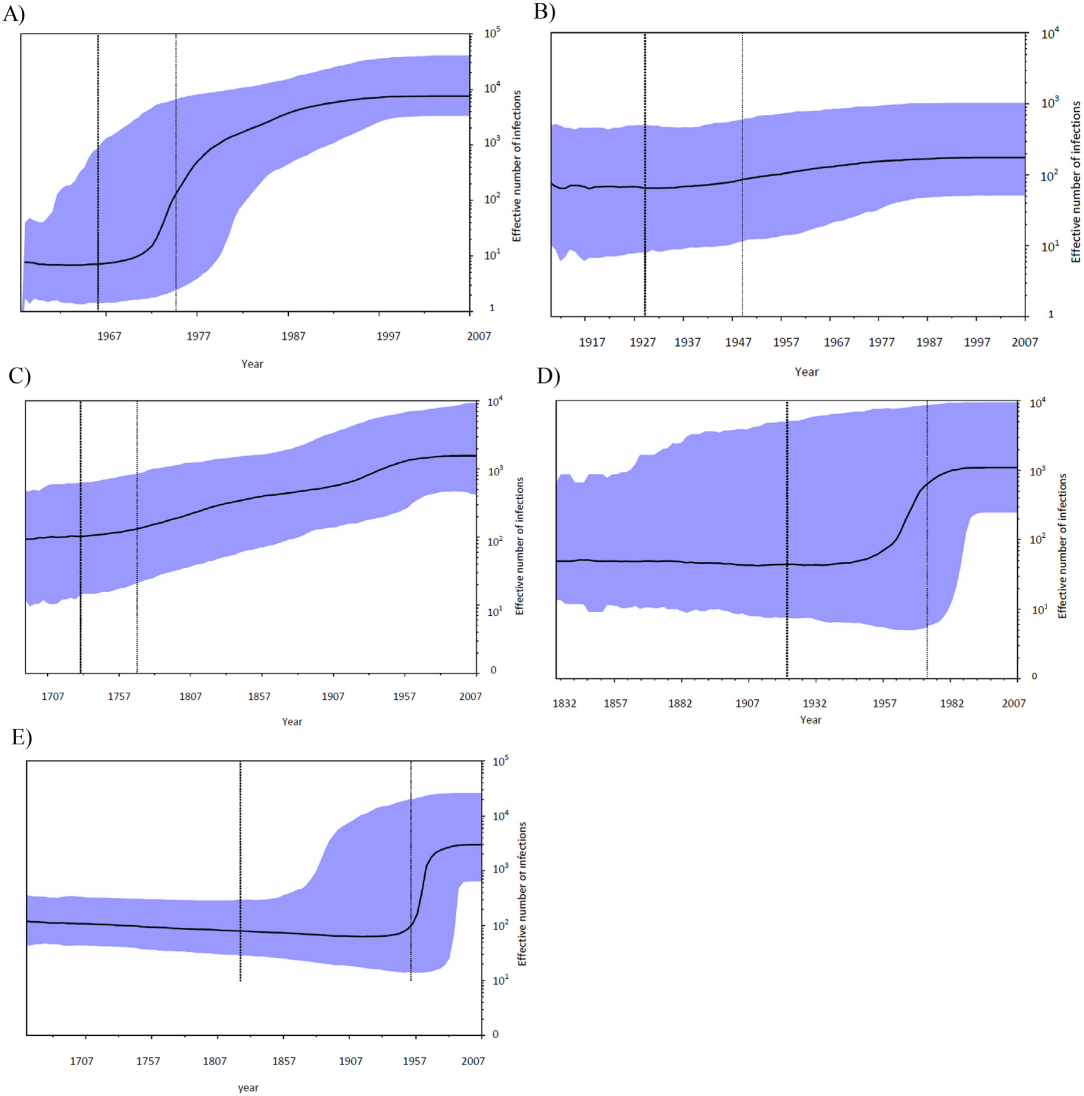
Bayesian Skyline Plots (BSPs) for strains of HCV subtypes. (A) Strains of subtype 1a, cluster A. (B) BSPs for subtype 1a, cluster B. (C) BSPs for subtype 1b, cluster C. (D) BSPs for subtype 3a, cluster D. (E) BSPs for subtype 3a, cluster E. The black lines are the estimates of the effective number of infections and the blue shaded areas indicate 95% credible intervals of effective number of infections. Black and grey dotted lines represent median estimation and lower bond of the 95% credible interval for the cluster age, respectively.

**Fig 6:**
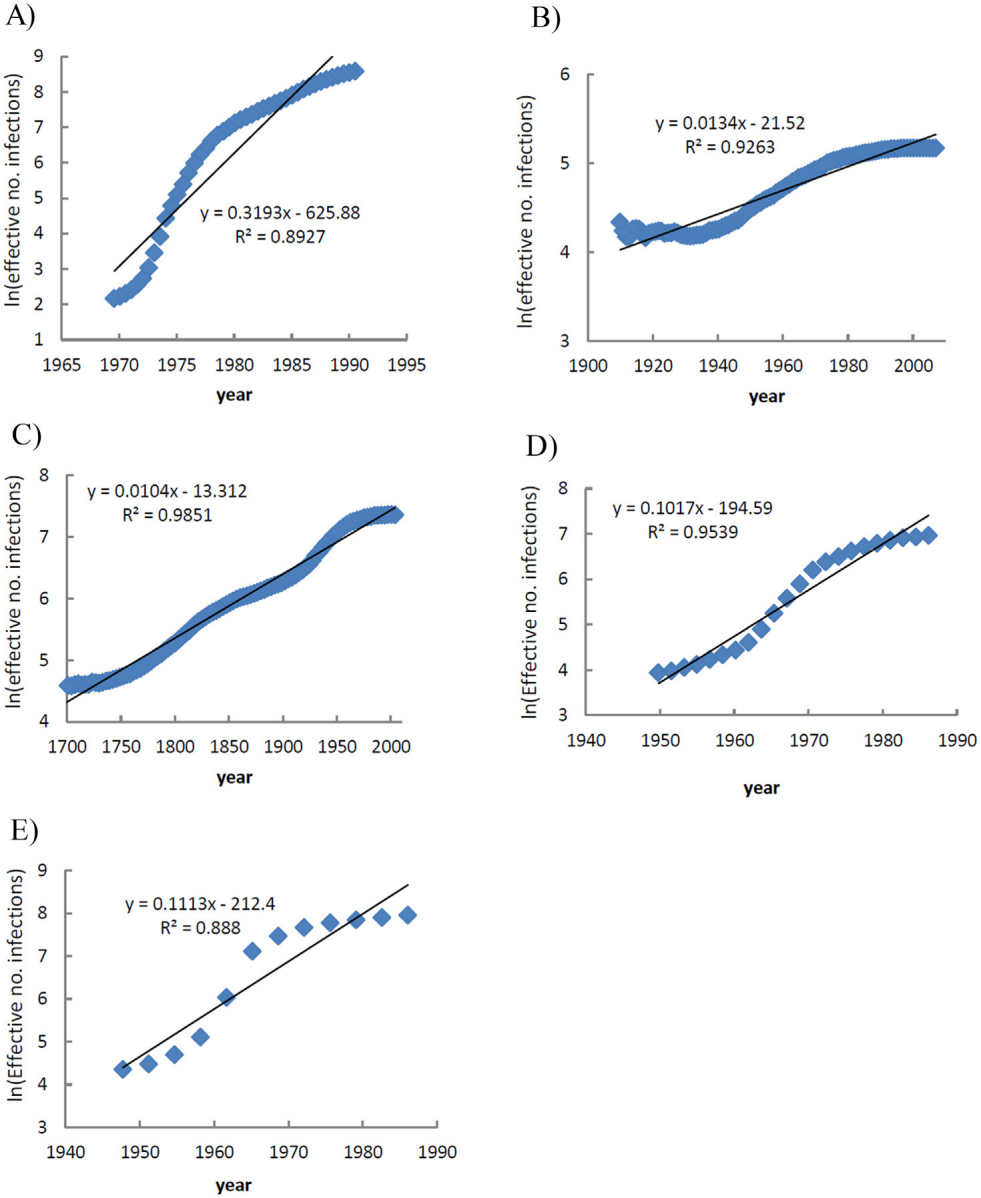
The mean value of exponential growth rates of HCV subtypes for which BSPs were calculated. (A) Subtype 1a, cluster A. (B) Subtype 1a, cluster B. (C) Subtype 1b, cluster C. (D) Subtype 3a, cluster D. (E) Subtype 3a, cluster E. The natural logarithm of the effective number of infections is plotted versus time period for which the corresponding BSP has a growing trend. The fitted regression equation to the natural logarithm of effective number of infections (as a dependent variable) and time (as an independent variable) is shown.

Five clusters A, B, C, D, and E were analyzed using BSP (Fig 5). As indicated for cluster A, the effective number of infections increased at a rapid exponential rate between 1970 and 1990 (mean exponential growth rate = 0.31 per year) (Figs 5A and 6A). The exponential growth of this cluster led to an approximate 43.5-fold increase in the effective number of infection compared to cluster B strains. The current effective population size of cluster A was higher than those of other clusters (Table 1). This finding is in accordance with the reports that show subtype 1a is the most prevalent type among Iranian patients with inherited bleeding disorders [5, 6]. The population dynamic of cluster B showed a period of constant, and very slow growth between 1910 and 2005 as well as the same mean growth rate (0.01 per year) as cluster C (Figs 5B and 6B). Fig 5C indicates the population dynamics of cluster C. The effective number of infections in this cluster increased at a constant and very slow rate for a long period of time. The mean exponential growth rate of cluster C between 1700 and 2000 was approximately 0.01 (Fig 6C). This cluster strains had a significantly older common ancestor compared to other clusters (Table 1). Our results suggest that the most recent ancestor of HCV subtype 1b among Iranian patients with inherited bleeding disorders existed around 300 years ago. Cluster D and E showed a population dynamic history in which the virus initially increased approximately exponentially from about 1950 to 1985 after which the growth rate slowed considerably in both clusters (Fig 5D and 5E). The mean exponential growth rates for clusters D and E were 0.10 and 0.11, respectively (Fig 6D and 6E). The estimated date of MRCA for cluster E was 1826 while this date for cluster D was 1922 (Table 1). The older common ancestor for cluster E is likely to contain fewer samples and more divergent strains than cluster D.

**Table 1:** Estimated evolutionary of each subtype (median and 95% confidence intervals).

Subtype	Date of MRCA year	Transition/transversion rate ratio (k)	Current effective no. infections	Rate heterogeneity parameter (α)		
				codon position 1	codon position 2	codon position 3
(1a) Cluster A	1967 (1958–1975)	18.50 (13.02–25.20)	7658 (3289–40675)	0.608 (0.341, 1.002)	0.399 (0.001, 1.336)	0.420 (0.001,1.32)
(1a) Cluster B	1930 (1910–1950)	9.46 (2.37–24.73)	176 (51–1033)	0.392 (0.001, 1.590)	0.539 (0.015, 1.83)	0.364 (0.002, 1.473)
(1b) Cluster C	1730 (1692, 1770)	12.85 (6.56–21.74)	1563 (422–9340)	0.372 (0.001, 1.577)	0.565 (0.126, 1.723)	0.260 (0.001, 1.317)
(3a) Cluster D	1922(1836–1974)	12.94 (5.57–24.30), 14.11 (6.40–25.11)	1099 (247–9428)	0.347 (0.001, 1.509)	1.031 (0.330, 2.178)	0.393 (0.001, 1.501)
(3a) Cluster E	1826 (1662–1954)	16.67 (7.81–30.79), 30.89 (14.24–55.21)	2975 (644–26237)	0.287 (0.001, 1.370)	1.176 (0.458, 2.331)	0.254 (0.001, 1.135)

## Discussion

The present study provides the first comprehensive phylogenetic analysis on HCV subtypes distribution among Iranian patients with inherited bleeding disorders. Prevalence and distribution of HCV subtypes among these patients were both different from IDUs but similar to the other transfusion related risk groups such as thalassemia and dialysis patients [5, 11, 20, 21]. The predominant subtype in IDUs has been reported to be 3a and the most prevalent subtype among the three transfusion risk groups has been found to be 1a that indicate the common routes of HCV transmission. Except for a limited number of hemophilia patients who had occasionally received untreated factor concentrates manufactured in European countries, all transfusion related risk groups in Iran have been treated with locally provided blood and blood products[4].

Moreover, the molecular analysis of the samples collected from patients with inherited bleeding disorders provided a picture of HCV epidemic history among these patients. Most of the samples analyzed belonged to subtypes 1a, followed by 3a. The rise of syringe availability, unsafe therapeutic injection, and increase in transfusion of blood and blood products (from the 1940s to the late 1990) coincide with the epidemic growth observed in both subtypes (1a and 3a) in this population. The increased number of opium using population (from 1940s to the mid 1950s) as well as the introduction of injection drug use in the 1960s in Iran are also highlighted in this phenomena[22, 23]. The higher growth rate of subtype 1a compared to 3a suggests the different efficiency of transmission routes. Indeed, the massive increase in parenteral iatrogenic procedures and drug use appears to have had strong impact in the expansion of HCV-1a and HCV-3a, respectively. These results are consistent with the previous reports from Iran that indicate the most prevalent HCV subtypes among transfusion related risk groups and injection drug users [5, 20, 24].

The highest current effective number of infection observed in the BSP analysis for HCV-1a support the view that this type is the youngest subtype among Iranian patients with inherited bleeding disorders. The time of the most recent common ancestor for subtype 1a strains was estimated to be 1930 (1910–1950), which is in line with the evidence obtained from molecular studies indicating the subtype 1a epidemic growth started ~100 years ago[25]. The reconstructed epidemic histories also showed a period of exponential growth between 1970 and 1990 among patients with inherited bleeding disorders. Although this period seems to be delayed one decade compared with the time of HCV-1a desperation in developed countries, this finding is in accordance with the studies that show subtype 1a has probably expanded from the developed nations to the developing countries[16, 17]. It is tempting to speculate that this subtype rapidly diffused to the Iranian general population through modern contact network such as specific iatrogenic procedures and injection drug use. Afterward, local epidemics among Iranian patients with inherited bleeding disorders were further established through donating infected blood by HCV-1a infected individuals or drug users particularly IDUs. Our analysis suggests that the exponential expansion of HCV-1a reached a plateau around 1996, which coincides with the implementation of anti-HCV screening and the use of virally inactivated factor concentrates. This finding is in agreement with studies reporting a sharp decline in the incidence of new HCV infections among hemophiliacs in Iran after 1996[26].

The epidemic growth for subtype 3a from 1950s among patients with inherited bleeding disorders probably coincided with a very high opium using population and unsafe therapeutic injections such as blood transfusion. Several studies have reported that in 1940s, the number of opium addict was around one and half million, considering the total population of 14 million[22]. This trend continued till 1955. In fact, it was in the mid 20^th^ century that opium became a serious concern in Iran, with one of the highest opium using population in the world[27]. Various reports indicate that non-IDU have had a higher prevalence of HCV than the general population. This prevalence was 10–20% in Europe, 5–30% in USA, 10.2% in Japan, and 35.1% in Brazil [28–31]. In Iran, the rate of HCV infection among non-IDUs outside and inside of prison was 8.2% and 29.1–36.7%, respectively [32–34].

It is more likely that subtype 3a could disseminate through drug use among Iranian general population. The beginning of subtype 3a epidemic growth among Iranian patients with inherited bleeding disorders coincided with the establishment of blood transfusion service in 1952. However, this epidemic growth kept sustained by IDU and infected blood products. In 1960, with introduction of new mode of drug administration (IDU) into Iran, the incidence of HCV-3a infection among drug users most likely increased[23]. In 1961, Red Lion and Sun Society started locally providing single blood donor products for hemophilia patients and the trend of exponential growth of this subtype maintained due to the transfusion blood and blood products provided by blood sellers that most of them were drug addict[1]. The exponential expansion of HCV-3a reached a plateau around 1985, which preceded that of subtype 1a by approximately 10 years. This finding suggests that subtype 3a has stronger link to IDU than subtype 1a. Indeed, it appears that decline in the level of drug use due to the first severe anti-drug campaign between 1980 and 1986 following the 1979 revolution led subtype 3a to reach a plateau[35]. However, at the beginning of the 1990s, the trend of this decline stopped and the rate of drug use reversed to its previous level in the early 1990s before reincreasing more rapidly from 1995. Since this time coincided with the screening of the blood donors for HCV, the effect of this rapid increase in the level of drug use, especially IDU, are not observed in data presented in this study.

Our results also showed that despite a decline in the level of drug use in the 1980s, the exponential growth of subtype 1a did not reach a plateau. This finding suggests that exponential increase in HCV-1a infection had stronger link to non-IDU transmission such as transfusion of blood and blood products. As such, HCV-1a continued its exponential increase up to 1996 when blood screening for HCV was implemented. This finding is in line with the reports that indicate HCV-1a is the most prevalent type in transfusion-related risk groups and subtype 3a is the most predominant type among IDUs in Iran.

Moreover, our finding suggests that the most recent ancestor of subtype 1b among patients with inherited bleeding disorders existed around 300 years ago. The epidemiological surveys show that the global spread of subtype 1b is largely due to its transmission through hemodialysis, blood transfusion and blood products[24]. This observation corroborates reports from Iran that indicate HCV-1b prevalence is much greater in transfusion related risk groups such as patients with hemophilia (14.4%),thalassemia (15%) and dialysis (6.9%) than that of IDUs (0.9%) [5, 11, 20, 21].

Subtype 1b is most likely to exist among Iranian general population in the past and transmitted by a variety of undefined social and domestic modes although its transmission has been more commonly associated with infected blood and blood products from ~1945 to ~1996. During the last century, HCV-1b growth rate (0.01) was considerably lower than those of HCV-1a (0.31) and 3a (0.1), which reinforces a consistent and low exponential rate for subtype 1b in this time frame. The low dissemination of subtype 1b observed among patients with inherited bleeding disorders in this study may be explained by several reasons:

1. We did not import blood and single blood donor products from Western countries between the 1940s and the 1980s, when this subtype grew exponentially through infected blood and blood products in these countries[25].
2. In Iran, only a small proportion of hemophilia patients were occasionally treated with non-virally inactivated factor concentrates between 1970 and 1986. During this period, most of hemophiliacs received locally produced cryoprecipitate and fresh frozen plasma.
3. Transmission of this subtype has been less commonly associated with drug use (especially IDU)[25]. This finding corroborates published reports indicating the low prevalence of subtype1b (0.9%) among Iranian IDUs [11, 20].

In conclusion, our phylogenetic analysis highlights the distribution of HCV subtypes among patients with inherited bleeding disorders in Iran. This analysis also provides a picture of HCV epidemic history and plausible transmission routes including infected blood products and drug use. Regardless of sharp decrease in HCV transmission, high rates of HCV infection in this risk group indicate the need for surveillance programs to prevent new HCV infections and further efforts to treat these patients, especially those with genotype 1, with new direct acting antiviral drugs to control current and future HCV related complications.

## References

[1] Azizi MH, Nayernouri T, Bahadori M. The history of the foundation of the Iranian National Blood Transfusion Service in 1974 and the Biography of its Founder;Professor Fereydoun Ala. Arch Iran Med. 2015; 18(6): 393–400. . 26058940

[2] Farhadi Langeroodi M, Eftekhari MA, Ahmadi J. Blood transfusion in Iran. in Principles of blood transfusion in medicine. Iran: Iranian Blood Transfusion Organization; 1998 pp. 27–55.

[3] Abolghasemi H, Maghsudlu M, Kafi-Abad SA, Cheraghali A. Introduction to Iranian blood transfusion organization and blood safety in Iran. Iran J Publ Health. 2009; 38(1): 82–87.

[4] Rezvan H, Abolghassemi H, Kafiabad SA. Transfusion-transmitted infections among multitransfused patients in Iran: a review. Transfus Med. 2007; 17(6): 425–433. 10.1111/j.1365-3148.2007.00794.x . 18067646

[5] Samimi-Rad K, Shahbaz B. Hepatitis C virus genotypes among patients with thalassemia and inherited bleeding disorders in Markazi province, Iran. Haemophilia. 2007; 13(2): 156–163. 10.1111/j.1365-2516.2006.01415.x . 17286768

[6] Keshvari M, Alavian S, Behnava B, Miri S, Tabatabaei S, Abolghasemi H, et al Distribution of hepatitis C virus genotypes in iranian patients with congenital bleeding disorders. Iran Red Crescent Med J. 2010; 2010(6): 608–614.

[7] Lu L, Nakano T, He Y, Fu Y, Hagedorn CH, Robertson BH. Hepatitis C virus genotype distribution in China: predominance of closely related subtype 1b isolates and existence of new genotype 6 variants. J Med Virol. 2005; 75(4): 538–549. 10.1002/jmv.20307 . 15714489

[8] Pybus OG, Barnes E, Taggart R, Lemey P, Markov PV, Rasachak B, et al Genetic history of hepatitis C virus in East Asia. J Virol. 2009; 83(2): 1071–1082. 10.1128/JVI.01501-08 ; PMCID: PMC2612398. 18971279 PMC2612398

[9] Nakano T, Lu L, He Y, Fu Y, Robertson BH, Pybus OG. Population genetic history of hepatitis C virus 1b infection in China. J Gen Virol. 2006; 87(1): 73–82. . 16361419 10.1099/vir.0.81360-0

[10] Pybus OG, Drummond A, Nakano T, Robertson B, Rambaut A. The epidemiology and iatrogenic transmission of hepatitis C virus in Egypt: a Bayesian coalescent approach. Mol Biol Evol. 2003; 20(3): 381–387. 10.1093/molbev/msg043 . 12644558

[11] Samimi-Rad K, Nategh R, Malekzadeh R, Norder H, Magnius L. Molecular epidemiology of hepatitis C virus in Iran as reflected by phylogenetic analysis of the NS5B region. J Med Virol. 2004; 74(2): 246–252. 10.1002/jmv.20170 . 15332273

[12] Darriba D, Taboada GL, Doallo R, Posada D. jModelTest 2: more models, new heuristics and parallel computing. ‎Nat. Methods. 2012; 9(8): 772–772. 10.1038/nmeth.2109 ; PMCID: PMC4594756. 22847109 PMC4594756

[13] Posada D. jModelTest: phylogenetic model averaging. Mol Biol Evol. 2008; 25(7): 1253–1256. 10.1093/molbev/msn083 . 18397919

[14] Guindon S, Dufayard JF, Lefort V, Anisimova M, Hordijk W, Gascuel O. New algorithms and methods to estimate maximum-likelihood phylogenies: assessing the performance of PhyML 3.0. Syst Biol. 2010; 59(3): 307–321. 10.1093/sysbio/syq010 . 20525638

[15] Guindon S, Gascuel O. A simple, fast, and accurate algorithm to estimate large phylogenies by maximum likelihood. Syst Biol. 2003; 52(5): 696–704. 10.1080/10635150390235520 . 14530136

[16] Yuan M, Lu T, Li C, Lu L. The evolutionary rates of HCV estimated with subtype 1a and 1b sequences over the ORF length and in different genomic regions. PLoS One. 2013; 8(6): e64698 10.1371/journal.pone.0064698 PMCID: PMC3675120. 23762247 PMC3675120

[17] Culasso AC, Elizalde M, Campos RH, Barbini L. Molecular survey of hepatitis C virus in the touristic city of Mar del Plata, Argentina. PLoS One. 2012; 7(9): e44757 10.1371/journal.pone.0044757 ; PMCID: PMC3454412. 23028605 PMC3454412

[18] Drummond AJ, Rambaut A. BEAST: Bayesian evolutionary analysis by sampling trees. BMC Evol Biol. 2007; 7(1): 214 10.1186/1471-2148-7-214 ; PMCID: PMC2247476. 17996036 PMC2247476

[19] Drummond AJ, Ho SY, Phillips MJ, Rambaut A. Relaxed phylogenetics and dating with confidence. PLoS Biol. 2006; 4(5): e88 10.1371/journal.pbio.0040088 ; PMCID: PMC1395354. 16683862 PMC1395354

[20] Samimi-Rad K, Toosi MN, Masoudi-nejad A, Najafi A, Rahimnia R, Asgari F, et al Molecular epidemiology of hepatitis C virus among injection drug users in Iran: a slight change in prevalence of HCV genotypes over time. Arch Virol. 2012; 157(10): 1959–1965. 10.1007/s00705-012-1369-9 . 22695769

[21] Samimi-Rad K, Asgari F, Nasiritoosi M, Esteghamati A, Azarkeyvan A, Eslami SM, et al Patient-to-patient transmission of hepatitis C at Iranian thalassemia centers shown by genetic characterization of viral strains. Hepat Mon. 2013; 13(1). 10.5812/hepatmon.7699 ; PMCID: PMC3622054. 23585766 PMC3622054

[22] Reid G, Costigan G. Revisiting The Hidden Epidemic: A Situation Assessment of Drug Use in Asia in the context of HIV/AIDS. 2002 100–108.

[23] mokri A. Brief overview of the status of drug abuse in Iran. 2002a; Available: www.ams.ac.ir/AIM/0253/0253184.htm

[24] Somi MH, Keivani H, Ardalan MR, Farhang S, Pouri AA. Hepatitis C virus genotypes in patients with end-stage renal disease in East Azerbaijan, Iran. Saudi J Kidney Dis Transpl. 2008; 19(3): 461 . 18445914

[25] Pybus OG, Charleston MA, Gupta S, Rambaut A, Holmes EC, Harvey PH. The epidemic behavior of the hepatitis C virus. Science. 2001; 292(5525): 2323–2325. 10.1126/science.1058321 11423661

[26] Samimi Rad K, Hosseini M, Asgari F, Alavian S, Tahaei M, Satari M. Hepatitis C virus infection among multi-transfused patients and personnel in haemodialysis units in central Islamic Republic of Iran. East Mediterr Health J. 2012; 18(3): 227–235. . 22574475 10.26719/2012.18.3.227

[27] Ahmadi J, Pridmore S, Alimi A, Cheraghi A, Arad A, Parsaeyan H, et al Epidemiology of opium use in the general population. Am J Drug Alcohol Abuse. 2007; 33(3): 483–491. 10.1080/00952990701301293 . 17613976

[28] Hagan H, Thiede H, Des Jarlais DC. HIV/hepatitis C virus co-infection in drug users: risk behavior and prevention. AIDS. 2005; 19(suppl 3): S199–S207. . 16251818 10.1097/01.aids.0000192090.61753.d4

[29] Quaglio G, Lugoboni F, Pajusco B, Sarti M, Talamini G, Lechi A, et al Factors associated with hepatitis C virus infection in injection and noninjection drug users in Italy. Clin Infect Dis. 2003; 37(1): 33–40. 10.1086/375566 . 12830406

[30] Wada K, Greberman SB, Konuma K, Hirai S. HIV and HCV infection among drug users in Japan. Addiction. 1999; 94(7): 1063–1069. . 10707444 10.1046/j.1360-0443.1999.947106311.x

[31] Oliveira-Filho AB, Sawada L, Pinto LC, Locks D, Bahia SL, Castro JA, et al Epidemiological aspects of HCV infection in non-injecting drug users in the Brazilian state of Pará, eastern Amazon. Virol J. 2014; 11(1): 1 10.1186/1743-422X-11-38 ; PMCID: PMC4077103. 24564954 PMC4077103

[32] Malekinejad M, Navadeh S, Lotfizadeh A, Rahimi-Movaghar A, Amin-Esmaeili M, Noroozi A. High hepatitis C virus prevalence among drug users in Iran: systematic review and meta-analysis of epidemiological evidence (2001–2012). Int J Infect Dis. 2015; 40: 116–130. 10.1016/j.ijid.2015.09.022 . 26460088 PMC8741151

[33] Amiri ZM, Rezvani M, Shakib RJ, Shakib AJ. Prevalence of hepatitis C virus infection and risk factors of drug using prisoners in Guilan province. East Mediterr Health J. 2007; 13(2): 250–256. . 17684845

[34] Mohammad Alizadeh A, Alavian SM, Jafari K, Yazdi N. Prevalence of hepatitis C virus infection and its related risk factors in drug abuser prisoners in Hamedan-Iran. World J Gastroenterol. 2005; 11(26): 4085–4089. 10.3748/wjg.v11.i26.4085 PMCID: PMC4502106. 15996035 PMC4502106

[35] Dalvand S, Agahi C, Spencer C. Drug addicts seeking treatment after the Iranian Revolution: a clinic-based study. Drug Alcohol Depend. 1984; 14(1): 87–92. 10.1016/0376-8716(84)90023-1 . 6489156

